# Development of 4T1 breast cancer mouse model system for preclinical carbonic anhydrase IX studies

**DOI:** 10.1002/2211-5463.70052

**Published:** 2025-05-15

**Authors:** Zane Kalniņa, Ilva Liekniņa, Svetlana Koteloviča, Ramona Petrovska, Gediminas Žvinys, Agne Petrosiute, Asta Zubrienė, Matīss Toms Laugalis, Vendija Skeltona, Juris Jansons, Madara Kreishmane, Edita Čapkauskaitė, Daumantas Matulis, Kaspars Tārs

**Affiliations:** ^1^ Latvian Biomedical Research and Study Centre Riga Latvia; ^2^ Faculty of Medicine and Life Sciences University of Latvia Riga Latvia; ^3^ Department of Biothermodynamics and Drug Design, Institute of Biotechnology, Life Sciences Center Vilnius University Lithuania

**Keywords:** 4T1 model, breast cancer, cancer imaging, carbonic anhydrase IX, hypoxia, preclinical models

## Abstract

Triple‐negative breast cancer (TNBC) is the most aggressive type of breast cancer, for which targeted treatment is currently lacking. Carbonic anhydrase IX (CAIX) is a known cancer target due to its selective overexpression in hypoxia, a hallmark of many solid cancers including TNBC. This study aimed to develop a robust murine TNBC cell line 4T1‐based model system that could be used in the comprehensive preclinical evaluation of targeting CAIX. The model is based on the original 4T1 breast cancer cell line and two genetically edited versions of it—one with biallelic CRISPR/Cas9‐mediated *Car9* inactivation and another with constitutively expressed *Car9*, thus ensuring negative and positive controls for CAIX production in the model system, respectively. The generated cell lines were validated for CAIX production and characterised functionally *in vitro* and *in vivo* after orthotopic implantation in syngeneic BALB/c mice. Results demonstrated significantly reduced primary tumour growth and metastatic progression rates in animals with CAIX‐deficient tumours, while the CAIX‐expressing tumours had vascularised phenotypes with prominent central areas of coagulative necrosis. The differential CAIX expression levels in the model were preserved during tumour growth in syngeneic mice, as verified by *in vivo* imaging using a novel high‐affinity CAIX‐specific near‐infrared (NIR) fluorescent imaging probe, GZ22‐4. Constitutive overexpression of autologous CAIX did not elicit specific autoantibody responses *in vivo*, demonstrating the suitability of this model for evaluating the efficacy of anti‐CAIX vaccination as a therapeutic strategy. The *in vivo* study was repeated as an independent experiment and demonstrated good robustness of the developed model.

## Graphical abstract

Carbonic anhydrase IX (CAIX) is a well‐recognised therapeutic target and prognostic biomarker in cancer. We developed and characterised a robust murine breast cancer model system that is suitable for CAIX studies *in vitro* and *in vivo*—it comprises both CAIX‐positive and CAIX‐negative controls and provides a solid platform for the comprehensive preclinical assessment of CAIX‐targeted therapeutic strategies or novel imaging agents.

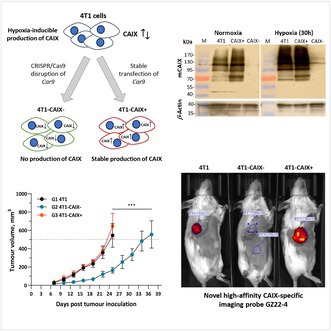

**Abbreviations**
*CA9*
: carbonic anhydrase 9 gene (human)
CAIX: carbonic anhydrase IX
*Car9*
: carbonic anhydrase 9 gene (murine)
Cas9: CRISPR‐associated protein 9
CMV: cytomegalovirus
CRISPR: clustered regularly interspaced short palindromic repeats
DAPI: 4′,6‐diamidino‐2‐phenylindole
DMSO: dimethyl sulfoxide
GLOBOCAN: Global Cancer Observatory
H&E: haematoxylin and eosin
IVC: individually ventilated cage
IVIS: *in vivo* imaging system
mCAIX: murine carbonic anhydrase IX
NIR: near‐infrared
pHe: extracellular pH
ROI: region of interest
SPF: specific pathogen‐free
TAA: tumour‐associated antigen
TNBC: triple‐negative breast cancer

According to GLOBOCAN, 2.3 million new breast cancer cases were diagnosed worldwide in 2020, making breast cancer the most frequently diagnosed cancer in both sexes (11.7%) overrunning lung cancer (11.4%) [1]. Triple‐negative breast cancer (TNBC) accounts for about 12–15% of all breast cancers, yet is the most aggressive subtype possessing high mortality, early and more frequent recurrence, and poor prognosis. It is a heterogeneous group of cancers, commonly characterised by the lack of oestrogen receptors (ER), progesterone receptors (PR), and it does not overexpress HER2 protein. TNBC currently lacks effective therapeutic targets, and the existing treatment strategies provide heterogeneous outcomes, thus clearly delineating a critical unmet clinical need [2].

One of the promising therapeutic targets of many solid tumours, including TNBC, is carbonic anhydrase IX (CAIX), elevated expression of which has been associated with worse prognosis in many cancer patients [3], including TNBC patients [4, 5]. CAIX is a transmembrane metalloenzyme with its catalytic site exposed in the extracellular space [6]. It catalyses a reversible hydration of carbon dioxide resulting in bicarbonate and protons [H_2_O + CO_2_ ↔ ${\mathrm{HCO}}_{3}^{-}$ + H^+^] [7]. *CA9* overexpression leading to elevated membranous CAIX levels is induced by hypoxia‐inducible factor 1 (HIF‐1) in the areas of oxygen deprivation in many solid tumours [8]. There CAIX ensures tumour cell escape from the highly acidic intracellular pH that results from glycolysis, the predominant form of cellular energy metabolism in hypoxia. As a result, the tumour microenvironment is acidified, which contributes to enhanced tumour cell migration, invasion, and metastases, rapid tumour growth, and deficient vascularisation [9, 10]. Importantly, CAIX has a limited expression in normal tissues—it is expressed predominantly in the gastrointestinal tract, mainly in gastric epithelial cells [11].

CAIX expression and function are evolutionarily conserved in vertebrates [12] and it serves the same function in humans and mice [13]. This favours the use of murine cancer models in preclinical CAIX studies due to their inherently high translational value. Indeed, several studies have exploited the well‐established 4T1 breast cancer mouse model [14], known to naturally overexpress the murine CAIX (mCAIX) in hypoxic areas at later stages of tumour development, to study the role of CAIX in cancer progression [15] or evaluate novel therapies targeting CAIX [16]. The 4T1 cells, when orthotopically transplanted in an immunocompetent syngeneic host, recapitulate the development of spontaneous metastatic human TNBC and yet represent fully murine disease [14].

This study aimed to develop a robust 4T1‐based murine TNBC model system that can be used in comprehensive preclinical evaluation of CAIX targeting strategy efficacy – namely, in this model system, true negative and positive controls for CAIX expression in 4T1 cells are introduced along the parental 4T1 cells by genetic manipulation of the *Car9* gene encoding for murine CAIX (mCAIX) that ensures its complete inactivation by CRISPR/Cas9 or stable expression under the CMV promoter, respectively. To the best of our knowledge, such a model system has not been developed before—most of the studies reporting on non‐clinical studies involving CAIX as a target have used shRNA‐mediated silencing of CAIX in cancer cells [17, 18, 19], including the 4T1 model [16]. Development and use of such a model system would be highly advantageous for the thorough evaluation of CAIX‐targeted therapies, especially immunotherapies and vaccination [20], and would represent an attractive tool for the evaluation of new CAIX‐targeted bioimaging agents [21, 22], as well as further understanding of the role of CAIX in the development and progression of TNBC. Here we describe the development of such a model system and characterise it for tumour development characteristics in the syngeneic immunocompetent host *in vivo* after orthotopic implantation.

## Materials and methods

### Genetic editing of 4T1 cell line

The commercially available mouse mammary adenocarcinoma cell line 4T1 (ATCC® CRL‐2539™) was used as the parental cell line in this study. To generate a 4T1 cell line stably expressing murine CAIX (designated as 4T1‐CAIX^+^), the coding sequence of *Car9* (CCDS18099.1) was cloned under the constitutive CMV immediate early promoter into *Xho*I/*Xba*I arms of the lentiviral expression vector pLVX‐Puro (Clontech, Takara Bio USA, Inc., Mountain View, CA, USA) (the service was provided by BioCat). For transfection efficacy control, the expression vector containing the coding sequence of GFP instead of *Car9* was used. Lenti‐X™ Packaging Single Shot (VSV‐G) (Takara Bio USA, Inc.) was used for the transfection of the lentiviral vector DNAs (i.e., GFP‐pLVX‐Puro and mCAIXst‐pLVX‐Puro) and lentivirus production in 293 T cells (ATCC® CRL‐3216™). The resultant preparations were quantified for the lentiviral particle amount by Lenti‐X GoStix Plus (Takara Bio USA, Inc. # 631280) and used for the vector DNA transfection into the 4T1 cells. The transfected clones were puromycin‐selected, and individual clones were isolated by using the limited dilution assay and cultured to establish monoclonal lines, in which stable expression of mCAIX was verified by western blot. Four of the clones with the highest CAIX production were pooled in equal proportions and used for orthotopic injection in the experimental animals.

For the allelic disruption of *Car9* in 4T1 cells, complementary RNA oligonucleotides that corresponded to the guide RNA sequence spanning the first exon of the gene (target sequence: AGAGGATCTATCGACTCCCGAGG corresponding to nucleotides 309–331 in the reference sequence NM_139305.2) were synthesised, hybridised, and cloned in the pX458 vector (*pSpCas9*(*BB)‐2A‐GFP*; Merck/Sigma‐Aldrich, Darmstadt, Germany, Addgene Plasmid #48138, Watertown, MA, USA) to generate an all‐in‐one CRISPR/Cas9 plasmid vector, which was transfected in the parental 4T1 cells by Lipofectamine 2000 reagent (Invitrogen, Thermo Fisher Scientific, Waltham, MA, USA). The transfection efficacy and Cas9 expression were validated by GFP production. Individual clones were further selected by using the limited dilution assay, cultured to establish monoclonal lines, and the *Car9* editing was confirmed by Sanger sequencing of genomic DNA (primers used for amplification: Car9‐Ex1‐Fwd 5′‐CTCCTTGGGAGACAGTTCATCT‐3′ and Car9‐Ex1‐Rev 5′‐ACCACTTACCTTTTTCATCCCC‐3′) and further validated for the level of mCAIX production by using western blot. The selected cell line was designated as 4T1‐CAIX^−^.

### Cell culturing and exposure to hypoxia

4T1, 4T1‐CAIX^−^ and 4T1‐CAIX^+^ cells were grown as an adherent monolayer in culturing medium DMEM with 4.5 g·L^−1^ glucose and UltraGlutamine I (Lonza #BE12‐604F/U1, Basel, Switzerland) supplemented with 100 μg·mL^−1^ Normocin™ (Invivogen #ant‐nr‐1) and 10% heat‐inactivated FBS (Sigma‐Aldrich #F7524). Cells were cultured at 37 °C and 5% CO_2_ equilibrated with atmospheric O_2_ in a humidified incubator. For the cell treatment with hypoxic conditions, the cells were seeded in the culturing medium and allowed to attach overnight, and then transferred to a humidified multi‐gas incubator (SANYO Electric Co., Osaka, Japan, #MCO‐5M) providing 1% O_2_, 94% N_2_ and 5% CO_2_, and incubated for 30 h. Cells from the same passage were cultured in parallel in normoxic conditions, and after 30 h cells from both hypoxic and normoxic conditions were used for downstream analyses.

### Determination of cell doubling time

Eighty thousand cells per well were plated in 6‐well adherent cell culture plates and grown in normoxic conditions. The viable cell number was estimated after 24 and 48 h by Trypan Blue staining and haemocytometer. The cell line doubling time was calculated by using the formula: doubling time = duration × ln(2)/ln (initial concentration/final concentration). The test was repeated as three independent experiments, and the mean values were expressed for comparison.

### Extracellular pH measurement

The extracellular pH (pHe) in the 4T1, 4T1‐CAIX^−^ and 4T1‐CAIX^+^ cell culture media was assessed based on the protocol described before [23]. Briefly, 3 × 10^5^ cells were plated in triplicates in 6‐well adherent plates and allowed to grow in the standard medium in normoxic conditions till confluence. Next, cells were washed with warm PBS and 3 mL of serum‐free IMDM medium without phenol red, supplemented with 25 nm HEPES and 2 mm L‐glutamine (Thermo Fisher Scientific #21056023) was added, and the cells were further incubated in parallel in normoxia and hypoxia for 30 h. The conditioned media were collected, and the cell debris was removed by centrifugation at 2000 ***g*** for 10 min. The pH of the media was immediately measured by a digital pH meter (Mettler‐Toledo, Greifensee, Switzerland), and the pH readouts were subtracted from the pH at time zero to determine changes in the pHe, which was further normalised to the number of live cells at the end of the experiment.

### Immunocytochemistry

The 4T1, 4T1‐CAIX^−^ and 4T1‐CAIX^+^ cells were seeded onto coverslips for adherent cells in 24‐well plates (1 × 10^4^ cells per well) and grown either in normoxia or hypoxia for 30 h as described above. After 30 h, cells were washed with PBS, fixed and permeabilised for 20 min with ice‐cold methanol/acetone (1:1 v/v), blocked (in 1% BSA, 0.1% Tween‐20, 22.52 mg·mL^−1^ glycine in PBS) for 45 min at RT, incubated with Anti‐Carbonic Anhydrase 9 antibody (1 : 100; Abcam #ab243660, Cambridge, UK) diluted in PBS with 1% BSA, 0.1% Tween‐20 overnight at 4 °C in a humidified chamber, washed 3 × 5 min in PBS, incubated with Alexa Fluor 488‐conjugated goat anti‐rabbit IgG secondary antibody (1 : 250; Thermo Fisher scientific #A11008) for 1 h at RT in the dark. After washing, the coverslips were mounted onto microscopy slides in ProLong™ Diamond Antifade Mountant with DAPI (Thermo Fisher Scientific #P36962) and assessed with fluorescent microscopy. For background control, cell staining with secondary antibody only was performed in all cell lines in parallel.

### Western blotting

To assess the mCAIX production level in the cell lines, the 4T1, 4T1‐CAIX^−^, and 4T1‐CAIX^+^ cells in the exponential growth phase were cultured in normoxia or hypoxia for 30 h as described above. After 30 h, the cells were washed twice with ice‐cold PBS and lysed in RIPA buffer (50 mm Tris, pH 8.0, 0.6 m NaCl, 4% Triton X‐100, 2% sodium deoxycholate, 0.1% SDS) containing Halt™ Protease Inhibitor Cocktail (ThermoScientific #78410). BCA Protein Assay (Pierce™ #23225) was used for total protein quantification. In total, 10 μg of total protein were suspended in Laemmli buffer without *β*‐mercaptoethanol to preserve the mCAIX dimer structure, separated by 10% SDS/PAGE in denaturing conditions, and electroblotted onto a nitrocellulose membrane. The membranes were blocked with 10% (w/v) skim milk powder (Millipore #70166, Darmstadt, Germany) in TBS buffer, and the mCAIX expression was detected by using recombinant Anti‐Carbonic Anhydrase 9/CA9 antibody (1 : 500; Abcam #ab243660). After stripping and repeated blocking, the membrane was probed with recombinant Anti‐*β*‐Actin antibody (1 : 1000; Abcam #ab115777) and visualised by using mouse anti‐rabbit IgG‐HRP secondary antibody (1 : 2000, SantaCruz #sc‐2357, Dallas, TX, USA) and ECL Select western Blotting Detection Reagents (GE Healthcare, Chicago, IL, USA) according to manufacturer's instructions. Relative quantification of CAIX signals normalised against *β*‐Actin expression was performed by using imagej software (USA) [24]. The experiment was repeated, and the obtained data from the experiments were used for the quantification of CAIX production.

### Animal care and *in vivo* experimental design

The experimental procedures in animals were approved by the National Animal Welfare and Ethics Committee (permit no. 130/2022) and performed in compliance with Directive 2010/63/EU as adopted in the national legislation. The *in vivo* procedures performed are described following the ARRIVE 2.0 guidelines to ensure transparent and comprehensive reporting.

An individual animal served as an experimental unit in both experiments and a fully randomised design was applied—mice were allocated to the study groups after stratified randomisation ensuring that animals from all groups are represented in each individual IVC. Power analysis was used to calculate the sample size by using G*power software [25, 26]. The sample size of seven animals per group was calculated by setting 90% power and α = 0.05, the published 4T1 tumour size variation [27], the expected effect size of stable CAIX expression and complete depletion of CAIX production, and the chosen statistical test for the primary outcome measure analyses. Two independent but identical experiments were performed 7 months apart—the first experiment served as an exploratory study to determine and compare the tumour growth dynamics and metastasis rate between three study groups (see Fig. 2A for the *in vivo* study design overview), and the second study served as a repeated study (this study was part of a bigger study, where these groups served as control groups for treatment groups [28]).

In total, 42 naïve adult SPF female BALB/cOlaHsd mice were obtained for study purposes from ENVIGO, Germany. The mice were purchased in two independent batches, 21 mice for each study. During the introduction, animals were randomly allocated in cages, five animals per cage. Individually ventilated cages (IVC) (Tecniplast #GM500, Milan, Italy), HEPA‐ventilated with SmartFlow air handling unit (Tecniplast) at 75 air changes per hour, were used for animal housing. Access to autoclaved water acidified to pH 2.5–3.0 with HCl and a phytoestrogen‐free maintenance diet for rodents (1320/TPF, Altromin, Lage, Germany) was provided *ad libitum*. Aspen wooden bedding, nesting material (Tapvei, Paekna, Estonia), cardboard tunnels (Velaz, Prague, Czech Republic) and aspen gnawing bricks (Tapvei) were provided in all cages, and cages were changed every 10 days. Animals were housed in an SPF facility with a controlled temperature of 24 ± 1 °C and relative humidity of 40–60%. Animal health monitoring was performed in line with FELASA recommendations [29].

### Syngeneic orthotopic breast cancer model

Animals underwent at least a 7‐day acclimatisation period before the procedure. At the age of 12 weeks, each animal was transplanted with syngeneic mouse breast adenocarcinoma cells (see Fig. 2). The cancer cell lines were tested negative for mycoplasma contamination (MycoSpy kit #M020, Biontex, Munich, Germany), grown to exponential growth phase, freshly harvested by 0.25% trypsin–EDTA solution, and washed three times with cold PBS and mixed with ice‐cold Matrigel® (Corning #356237, Corning, NY, USA) in a 1 : 1 (v : v) ratio immediately before injection. In total, 7 × 10^3^ live cells in 50 μL volume were injected into the third right mammary fat pad of 1.5% isoflurane‐anesthetised animals by using a 29G needle. The thoracic mammary gland area was depilated in advance to ensure the precision of the injection. Starting from day five post‐implantation, every third day, mice were weighed, and primary tumours were measured with a digital Vernier calliper in a blinded fashion. The tumour volume was calculated using the formula *V*_T_ = (*L* × *W* ^2^)/2, where *V* stands for tumour volume, *L*—length, and *W*—width. For the repeated (*R*) study, the same protocol was strictly followed—it was performed in the same animal facility, by the same personnel and mouse subline, thus providing information on the repeatability of the model.

Different endpoints were set for the two experiments (Fig. 2A; Table 1)—in the exploratory study, animals from each group were sacrificed when the average tumour volumes in the group reached a volume of ~ 600 mm^2^, while in the repeated study, which was part of an independent therapeutic study [28], the tumours were allowed to grow until they reached the volume of ~ 1688 mm^2^, which is in line with relevant guidelines [30]. Animal health was daily monitored for possible signs of suffering, and a suffering score sheet [31] was used to ensure a humane endpoint. Animals were humanely euthanized—under deep surgical isoflurane anaesthesia (5%), animals underwent cardiac puncture to collect terminal blood samples. Tissues of interest were collected and fixed in 4% buffered formaldehyde, and spleen lengths were measured. Tumours were extirpated, weighed, and measured in three dimensions. A modified ellipsoid formula *V*_T_ = *L* × *W* × *H* × π/6 was used for tumour volume calculation. Lungs were examined for the presence and size of metastatic nodules, which were measured and nodules < 1 mm and < 2 mm in diameter were regarded as small and medium, respectively, while those of > 2 mm in diameter were classified as large.

**Table 1:** Comparison of the *in vivo* model system study macroscopic findings. 4T1‐CAIX^−^, 4T1 cell line with no mCAIX production; 4T1‐CAIX^+^, 4T1 cell line with constitutive mCAIX production; CI, confidence interval; D, day; LN, lymph node.

Study groups	Group 1 (4T1)	Group 2 (4T1‐CAIX^–^)	Group 3 (4T1‐CAIX^+^)
Exploratory study			
Endpoint day:	D25/26	D39	D25/26
Time to tumour volume 500 mm^3^, days (median, 95% CI)	23.8 (23.1–30.1)	35.9 (31.8–39.0)	23.0 (22.5–27.0)
Tumour ulceration at endpoint	4/7	5/7	4/7
Lung metastases			
Macroscopic^a^	0/7	6/7	0/7
Microscopic	7/7	7/7	7/7
Other metastases^a^	0	0	0
Spleen size (mean ± SD)	26.7 ± 2.0	21.4 ± 1.6	26.7 ± 2.1
Repeated study			
Endpoint day:	D37/38	D36/37	D30/31
Time to tumour volume 500 mm^3^, days (median, 95% CI)	24.5 (21.2–27.2)	32.0 (29.6–35.3)	23.0 (21.0–25.5)
Tumour ulceration at endpoint	6/7	3/7	5/7
Lung metastases^a^	7/7	6/7	6/7
Small nodules	1/7	1/7	3/7
Medium nodules	3/7	4/7	1/7
Large nodules	3/7	1/7	2/7
Other metastases^a^	Axillary LN (1/7)	Sternum (1/7)	Sternum (1/7), Axillary LN (1/7), distal mammary gland (1/7)^b^
Spleen size, mm (mean ± SD)	30.1 ± 3.3	23.0 ± 2.4	32.0 ± 1.8

^a^ Data from necropsy examination. Nodules more than ~ 1 mm in diameter were defined as medium, and those more than ~ 2 mm as large.

^b^ The observed metastases are from different animals.

### Biofluorescent imaging of mCAIX expression *in vivo*

Biofluorescent imaging of mCAIX expression was done by using a novel high‐affinity CAIX‐specific near‐infrared (NIR) fluorescent imaging probe GZ22‐4 bearing a benzenesulfonamide head and an IR‐783 fluorophore. The chemical synthesis and characterisation of GZ22‐4 have been described in detail elsewhere [32]. The IUPAC name of GZ22‐4:

Sodium4‐((Z)‐2‐((E)‐2‐(2‐(4‐(43‐((2‐(cyclooctylamino)‐3,5,6‐trifluoro‐4‐sulfamoylphenyl)sulfonyl)‐3,41‐dioxo‐7,10,13,16,19,22,25,28,31,34,37‐undecaoxa‐4,40‐diazatritetracontyl)phenoxy)‐3‐((E)‐2‐(3,3‐dimethyl‐1‐(4‐sulfonatobutyl)‐3H‐indol‐1‐ium‐2‐yl)vinyl)cyclohex‐2‐en‐1‐ylidene)ethylidene)‐3,3‐dimethylindolin‐1‐yl)butane‐1‐sulfonate. Prior to the injection, lyophilised compound GZ22‐4 was stored at +4 °C in the dark and reconstituted in 100.1 μL DMSO to a final concentration of 10 mm.

On day 27 after the tumour cell injection, one animal from each group without obvious signs of tumour ulceration was selected for *in vivo* biofluorescent imaging to assess the mCAIX production level. The skin area on and around the tumour area, chest, and abdomen of the mice was depilated 24 h before imaging, and each animal was administered a single dose (100 μl) of 1 mm (4.65 mg·kg^−1^) GZ22‐4 in 50% Kolliphor® EL‐PBS solution through the lateral tail vein by using a 29 G needle. Biofluorescent images were taken at 24 and 48 h post‐injection – the animals were anaesthetized with isoflurane and imaged with the IVIS Spectrum imaging device (PerkinElmer #124262) to capture the epifluorescent signal (excitation 745 nm, emission 800 nm) according to the manufacturer's instructions and quantified by Living Image software Version 4.5.5.19626 (PerkinElmer, Waltham, MA, USA). The signal intensity was expressed as average radiance (p/s/cm^2^/sr) from the selected identical regions of interest (ROI; see Fig. 3B).

### Assessment of anti‐mCAIX autoantibody induction

The serum samples were obtained from cardiac puncture‐derived total blood samples by using the standard procedure and stored at −20 °C. ELISA plates were coated with recombinantly expressed mCAIX protein catalytic domain (1 μg per well, expressed in *P. pastoris* and purified as described earlier [33]) in coupling buffer overnight at 4 °C, washed, blocked in 1% BSA in PBS for 30 min at 37 °C, incubated with serially diluted serum samples in triplicates in 0.5% BSA, 0.05% Tween‐20 in PBS for 1 h at 37 °C. After washing, plates were incubated with a rabbit anti‐mouse IgG‐HRP conjugate (1 : 5000, Sigma‐Aldrich #A9044) for 1 h at 37 °C, washed, and the signal intensity visualised spectrophotometrically at 492 nm by using p‐phenylenediamine dihydrochloride (PPD) detection.

### Histological analyses

The histological analyses were performed according to a standard protocol described elsewhere [34]. Briefly, tissue samples of interest no more than 5 mm thick were fixed in 4% formaldehyde for 48 h immediately post‐mortem, dehydrated, and embedded in Paraplast Plus® (Sigma‐Aldrich). Tumour and lung tissue samples were cut in 5 μm slices with a microtome, deparaffinised, dehydrated, and stained with Mayer's haematoxylin (the step included blueing with 0.1% ammonium hydrochloride) and eosin, and assessed in light microscopy.

### Statistical analyses

The statistical analyses were performed with graphpad prism 9.5.0 (GraphPad, Boston, MA, USA). The normality of the obtained data distribution was assessed by using the Shapiro–Wilk test. For normally distributed data, ANOVA was used for multiple group comparison, followed by Tukey multiple comparison adjustment. For the data that did not follow the normal distribution, the nonparametric one‐way repeated‐measures Mann–Whitney test was used to compare data between any of the two study groups, while the Kruskal–Wallis test was used for multiple group comparison followed by Dunn's multiple comparison adjustment. *P*‐values of ≤ 0.05 were considered statistically significant. The nonparametric Spearman correlation rank test was performed for tumour and spleen parameter analyses.

## Results and Discussion

### Murine CAIX‐specific genetic editing of 4T1 cells

**Fig. 1:**
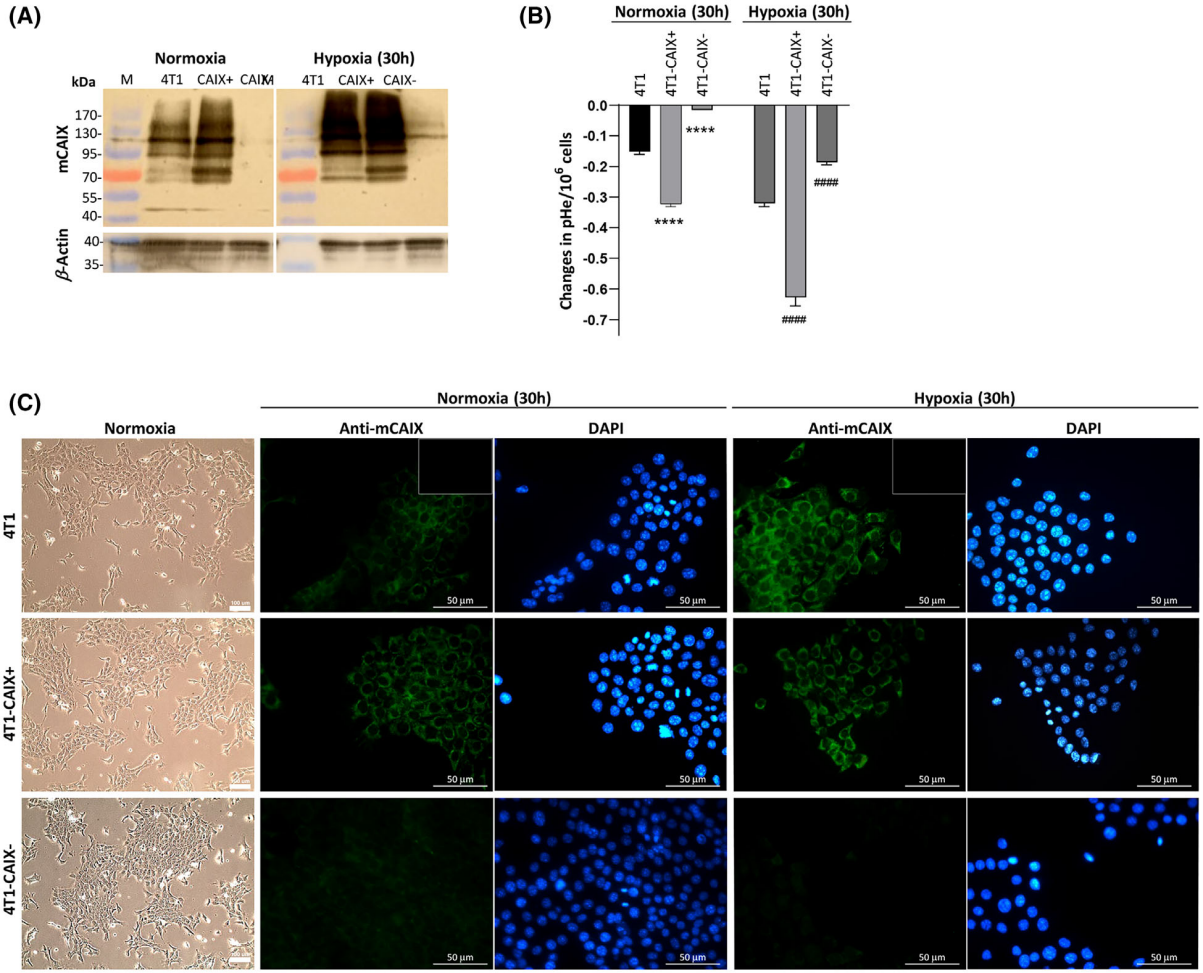
*In vitro* characterisation of the 4T1 cell lines edited for mCAIX expression. (A) Western blot results showing the level of mCAIX protein production in the generated 4T1 cell lines after 30 h incubation in normoxia and hypoxia (exposition time for mCAIX – 6 min, *β*‐Actin – 1 min). (B) Changes in extracellular Ph (pHe) after 30 h incubation in serum starvation conditions in normoxia and hypoxia, relative to baseline at 0 h, normalized to cell number (10^6^ cells) (*n* = 3, mean, SD); ANOVA multiple comparison‐adjusted *P‐*value: ****< 0.0001 Relative to 4T1 in normoxia, ^####^< 0.0001 Relative to 4T1 in hypoxia. (C) The left block shows the 4T1 cell line variant morphology in inverted phase contrast light microscopy (scale bar—100 μm) and the right block – Immunocytochemical detection of mCAIX production (green) side by side with DAPI staining of nuclei (blue) in the generated 4T1 cell line variants after 30 h incubation in normoxia and hypoxia (scale bar—50 μm; small upper left sections – Secondary antibody control). No nonlinear adjustments have been made in the original microphotographs; all have been acquired by using the same settings. 4T1‐CAIX, 4T1 cell line with no mCAIX production; 4T1‐CAIX^+^, 4T1 cell line with constitutive mCAIX production; *Car9*, murine carbonic anhydrase IX gene; mCAIX, murine carbonic anhydrase IX; M, size marker.

The commercially available 4T1 mouse mammary adenocarcinoma cell line closely resembles TNBC in humans and is known to overproduce mCAIX in hypoxic conditions [15]. In this study, the 4T1 cells were used as the parental cell line for genetic editing of the mCAIX encoding gene *Car9* to ensure either its complete knock‐out (designated as 4T1‐CAIX^–^) or constitutive production of CAIX irrespective of oxygen availability (4T1‐CAIX^+^) to provide the negative and positive controls for the mCAIX production in the model system, respectively. Full *Car9* knock‐out was achieved by using CRISPR/Cas9—cells were subcloned to monoclonality, *Car9* editing was assessed by Sanger sequencing, and a single selected cell line with both *Car9* alleles edited was validated for the lack of CAIX production by western blot and immunocytochemistry (Fig. 1A,C, respectively). To enable constitutive CAIX production, lentivirus‐mediated gene transfer was used to transfect the *Car9* gene under the CMV promoter in the 4T1 cell genomes. The edited cells were subcloned to monoclonality, and four individual clones producing the highest mCAIX levels based on the western blot results were selected for further tests and *in vivo* study, where they were pooled in equal proportions before injection in study animals.

In the western blot, the covalently bound dimer structure of mCAIX found on the plasma membrane is preserved due to the absence of reducing reagent in lysis buffer; the bands correspond to MW of ~ 94.6–116 kDa (Fig. 1A) depending on its posttranslational modification status. In normoxic conditions, 4T1 cells produced baseline amounts of mCAIX, which was significantly elevated irrespective of the oxygen availability in the 4T1‐CAIX^+^ cells (Fig. 1A,C)—in normoxia, 4T1‐CAIX^+^ cells produced on average 1.9 times more mCAIX in comparison to the parental 4T1 cells. In hypoxic conditions, the 4T1 cells and 4T1‐CAIX^+^ cells demonstrated on average 2.85 and 2.92 times higher mCAIX production in comparison to 4T1 cells in normoxia, when normalised to *β*‐Actin expression, respectively, due to the HIF‐1 induced overexpression of the *Car9* gene. In the case of 4T1‐CAIX^+^ cells, the demonstrated net mCAIX production increase in hypoxia represents the sum from the HIF‐1‐inducible expression added to the constitutive expression operating under the CMV promoter. There was no remarkable mCAIX production in either normoxic or hypoxic conditions in 4T1‐CAIX^−^ cells (Fig. 1A,C).

### *In vitro* characterisation of morphology and growth rate of CAIX‐edited 4T1 cells

When cultivated as adherent culture *in vitro*, the 4T1‐CAIX^−^ and 4T1‐CAIX^+^ cells exhibited the same morphology as the parental 4T1 cells after examination at the exponential growth phase in light microscopy (Fig. 1C, left panel). The cells did not show significant differences in doubling time when cultivated in standard normoxic conditions – for 4T1 cells it was 16.7 h (±1.3 h), for 4T1‐CAIX^−^ – 17.9 h (±1.6 h) and for 4T1‐CAIX^+^ – 16.3 (±1.4 h); one‐way ANOVA *P* = 0.42. Similar growth rates were observed in hypoxia – for 4T1 cells it was 16.1 h (±0.7 h), for 4T1‐CAIX^−^ – 17.7 h (±1.3 h) and for 4T1‐CAIX^+^ – 17.5 (±0.48 h); one‐way ANOVA *P* = 0.23.

### CAIX expression correlates with extracellular acidosis *in vitro*

Since one of the hallmarks in the cancer microenvironment is lowered extracellular pH while keeping the intracellular pH at an alkaline range [35], and the activity of CAIX is shown to be a critical determinant of this physiological process [18], we wanted to verify the correlation of mCAIX expression in the generated cell lines with the extracellular acidosis in normoxic and hypoxic conditions. As shown in Fig. 1B, when confluent cells were incubated in parallel in normoxic and hypoxic conditions for 30 h, significant acidification of the serum‐free HEPES‐buffered conditioned media of 4T1‐CAIX^+^ cells was observed relative to the parental 4T1 cells, while the opposite was demonstrated for the 4T1‐CAIX^−^ cells. In hypoxic conditions, parental 4T1 cells, due to the induction of CAIX production (Fig. 1A) lowered the pHe to twice the level detected in normoxia. The 4T1‐CAIX^+^ and 4T1‐CAIX^−^ cells closely resembled the effect on pHe that was observed in normoxia (Fig. 1B)—i.e., the reversal of extracellular acidosis by 4T1‐CAIX^−^ cells thus clearly demonstrates the functional effect of the CAIX expression on the generated breast cancer cells.

### mCAIX‐deficient tumours show significantly slower growth *in vivo*

**Fig. 2:**
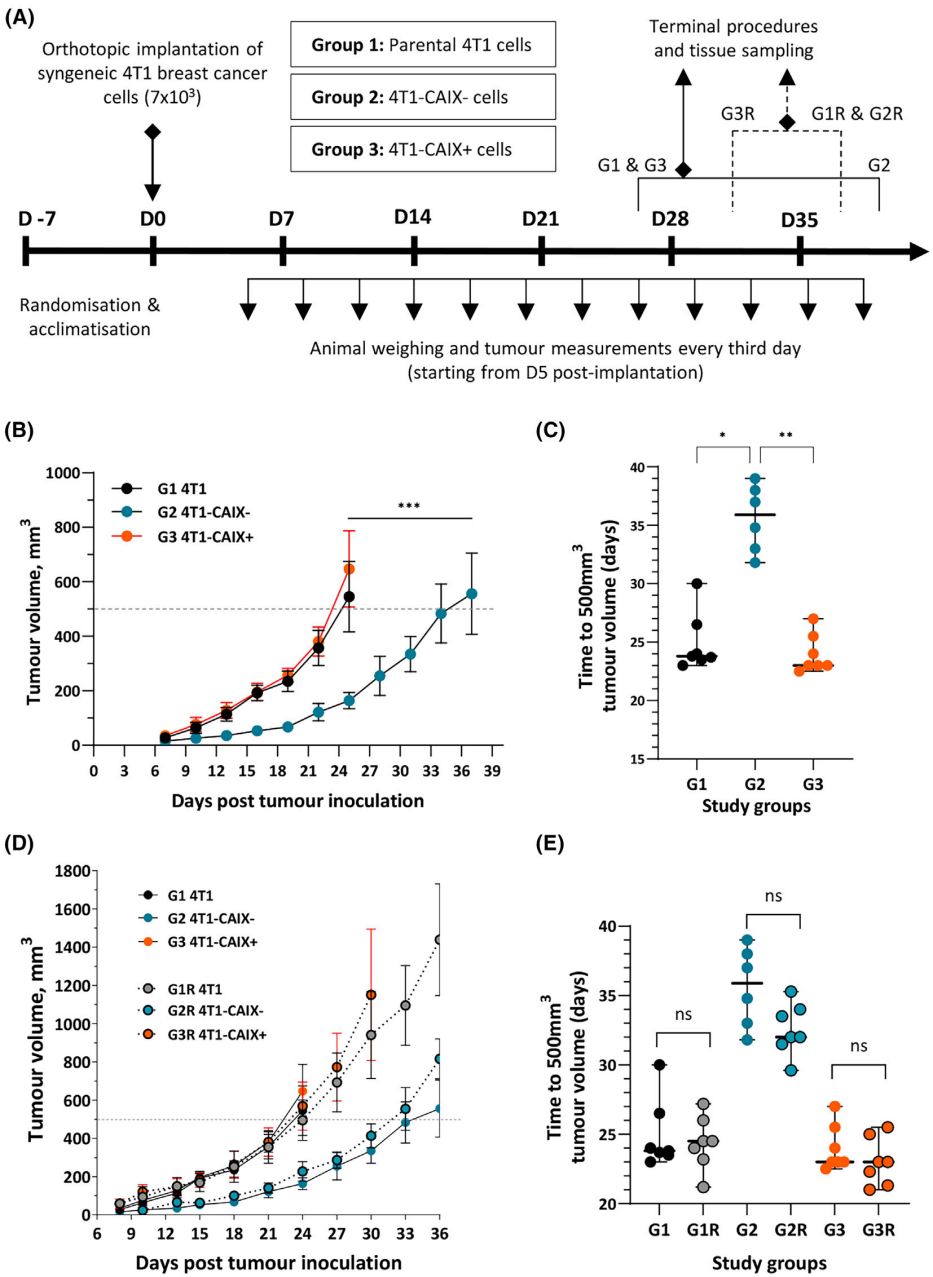
The *in vivo* study design and tumour growth comparison in mice bearing 4T1 tumours with differential mCAIX expression. (A) An overview of the *in vivo* study design. In the exploratory study, adult BALB/c female mice (*n* = 7) were orthotopically implanted with 4T1 murine breast cancer cells in 50% Matrigel and monitored for tumour growth dynamics. Depending on the study group, animals received either parental 4T1 cells (group 1) or 4T1‐CAIX^−^ (group 2), or 4T1‐CAIX^+^ (group 3) cells. This exploratory study was repeated as an independent procedure 7 months later by using another set of BALB/c female mice (*n* = 7); the terminal procedure timing is depicted with the dotted line in this case. (B) Comparison of the tumour growth dynamics between study groups (*n* = 7, mean values within the groups ± SD are shown). One‐way repeated‐measures Mann–Whitney test *P*‐value shown (group 1 and 2 data compared). (C) The time needed to reach tumour volume of 500 mm^3^ (median, 95% CI), dots represent individual animals (*n* = 7); Kruskal–Wallis multiple comparison‐adjusted *P*‐values shown. The data from a repeated study were compared to that from the exploratory study – (D) shows the comparison of tumour growth dynamics (*n* = 7; mean, SD) and (E) shows comparison of time needed to reach tumour volume of 500 mm^3^ (*n* = 7; median, 95% CI; Mann–Whitney test *P‐*values shown). 4T1‐CAIX^−^, 4T1 cell line with no mCAIX production; 4T1‐CAIX^+^, 4T1 cell line with constitutive mCAIX production; *Car9*, gene encoding for murine carbonic anhydrase IX; D, day; G, group; R, replicate; *P < 0.05, **P < 0.01, ***P < 0.001, ns – non‐significant.

To evaluate the characteristics of the tumour development *in vivo*, the generated 4T1‐CAIX^−^ and 4T1‐CAIX^+^ cell lines, along with the parental 4T1 cell line, were transplanted into syngeneic hosts to evaluate their tumourigenicity, tumour growth dynamics, and metastatic potential. Briefly, adult female BALB/c mice were orthotopically injected with mycoplasma‐free 4T1 cells in Matrigel—either parental or modified, and primary tumour size and animal weight were measured every third day. This exploratory study was repeated as an independent study to evaluate the robustness and repeatability of the *in vivo* model system. The overview of the study design and the set study endpoints are summarised in Fig. 2A (see also Table 1).

Exploratory study showed that in all study groups, tumour take rate was 100%. 4T1‐CAIX^−^ tumours revealed a significantly slower growth rate in comparison to CAIX‐expressing tumours (4T1‐CAIX^−^ versus 4T1 Mann–Whitney mean rank difference: −7, *P* = 0.00058; Fig. 2B). However, constitutive mCAIX expression did not provide a significant growth advantage over the parental 4T1 cells (Mann–Whitney mean rank difference: 2.1, *P* = 0.38). As an outcome measure, the time needed to reach the median tumour volume of 500 mm^3^ within a group was determined – 4T1 tumours required on average 23.8 days (95% CI: 23–30), 4T1‐CAIX^+^ tumours – 23.0 days (95% CI: 22.5–27.0) while for 4T1‐CAIX^−^ tumours it took significantly longer to develop tumours of the same size – 35.9 days (95% CI: 31.8–39.0), which is on average ~ 12.5 days longer (corresponding to ~ 35%; Kruskal–Wallis test *P* = 0.0002; Fig. 2C; Table 1). When the study was repeated, the obtained data showed a good coherence with that from the exploratory study (Fig. 2D,E; Table 1) demonstrating the robustness of the developed *in vivo* model system.

Several studies have reported on the effects of shRNA‐mediated knock‐down or CRISPR/Cas9‐mediated knock‐out of CAIX in various cancer cell lines. In most of the studies, shRNA‐mediated knock‐down of CAIX has been performed, including different human breast cancer cell lines UFH‐001 [17], MCF10A [18], MCF7, and MDA‐MB‐321 [19] as well as the murine 4T1 cells [16] chosen for this study, while its complete allelic disruption by CRISPR/Cas9 has been described in human colon adenocarcinoma cells LS174 [36] and human breast cancer cell lines MDA‐MB‐231 and T47D [19]. Results from all the studies are in agreement that perturbed CAIX expression leads to significantly decreased tumour cell proliferation and growth *in vivo*, which is in line with our study findings. Importantly, the shRNA‐mediated knock‐down of CAIX has been shown to control for mRNA transcript availability in hypoxia only partially [19, 36] thus, complete allelic disruption by CRISPR/Cas9 is a more reliable method to ensure a true negative control within a model system if one is required [37].

Lau *et al*. have reported the effects of the shRNA‐mediated mCAIX knock‐down on 4T1 tumour growth *in vivo* [16]. The study showed that the depletion of mCAIX in 4T1 cells resulted in drastic growth and metastasis rate reduction *in vivo* after orthotopic injection in a syngeneic host and had a significant effect on the overall survival of the mice—mice survived for 100 days after injection of 10^6^ 4T1 cells with CAIX knock‐down. In comparison to the data obtained in our study, the complete *Car9* knock‐out by CRISPR/Cas9 did not result in such a profound anti‐tumoural effect *in vivo*, although the overall effect of CAIX production loss on tumour progression was the same—the tumours developed significantly slower and demonstrated halted metastatic progression rates (Fig. 2B–E, Table 1). *In vivo* studies are inherently prone to variation and known as well as unknown biases affecting the study results. It was not possible to compare most of the variables in the performed procedures and thus further discuss the found discrepancies. Noteworthy, it has been shown that many factors including the substrain and the microbiological status of the used experimental animals, known and unknown confounding factors, type of study design, the tumour cell administration technique and route as well as the method chosen for gene silencing all may significantly affect the results and reproducibility of the results [38, 39]. Yet, the effect of mCAIX knock‐out in our study was highly repeatable (Fig. 2D,E) thus strengthening the internal validity of the model system and credibility of the obtained data.

### Tumour‐associated mCAIX production levels are preserved during 4T1 tumour variant growth *in vivo*

**Fig. 3:**
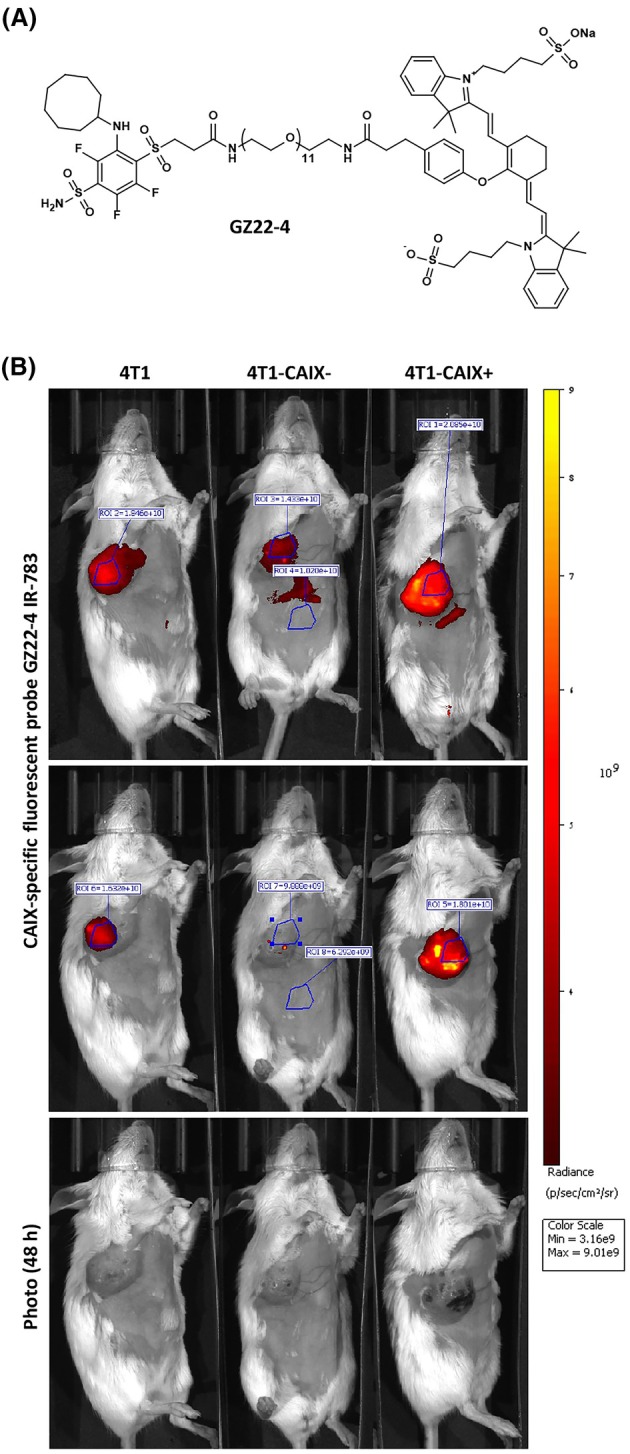
*In vivo* epifluorescence imaging of murine CAIX in tumour‐bearing animals. (A) The chemical structure of the novel high‐affinity CAIX‐specific near‐infrared fluorescent imaging probe GZ22‐4 used for *in vivo* imaging. (B) Epifluorescence imaging results of tumour‐bearing animals at day 27 post tumour cell implantation. Results demonstrate fluorescent signals detected in living animals by using the IVIS Spectrum *in vivo* imaging system 24 (upper panel) and 48 h (middle panel) after intravenous administration of the GZ22‐4 probe. Results are displayed in a yellow hot logarithmic scale. Grayscale photographs demonstrating the tumour size differences are shown below for comparison. 4T1‐CAIX^−^, 4T1 cell line with no mCAIX production; 4T1‐CAIX^+^, 4T1 cell line with constitutive mCAIX production; ROI, region of interest.

To validate the mCAIX production levels in the developed model system, at day 27 post tumour cell implantation, *in vivo* epifluorescence imaging of tumour‐bearing animals was performed after the injection of a novel CAIX‐specific NIR imaging agent GZ22‐4 (Fig. 3A), which specifically detects the cell surface expression of CAIX [32]. The 4T1 tumour demonstrated a centrally localised signal for mCAIX corresponding to the hypoxic area of the primary lesion—as seen from the signal in the 4T1 tumour, CAIX production in the more oxygenated peripheral areas is reduced. After a 48‐h period, the background‐subtracted average radiance value from the designated region of interest (ROI, Fig. 3B) in the 4T1‐CAIX^+^ tumour was 3.26 times stronger and in the 4T1 tumour—2.79 times stronger than in the tumours lacking CAIX production. The signal‐to‐noise ratio was best 48 h after the probe injection where the average radiance signal from the 4T1‐CAIX^−^ tumour did not exceed much that of the background signal (Fig. 3B), which is in agreement with the western blot results (Fig. 1B). Taken together, the obtained results confirmed that the differential CAIX production levels in the three cell lines used in the model system are preserved *in vivo*.

The fact that CAIX is almost undetectable in healthy tissues except for gastric mucosa [11] favours the use of CAIX as a promising target for solid tumour imaging [22]. The developed model system is optimal in evaluating new CAIX‐targeted imaging agent characteristics due to the differential CAIX production. Besides, the close proximity of the orthotopically developing 4T1 tumours to the body surface allows for easier acquisition of signals from *in vivo* epiluminescent and epifluorescent imaging, more often available in research infrastructures. Our *in vivo* pilot study showed that compound GZ22‐4 is highly specific for CAIX‐expressing tumours. Such results are consistent with the previously investigated *in vitro* measurements [32] demonstrating high affinity and selectivity of the compound for recombinant human CAIX. GZ22‐4 exhibited high affinity (*K*_d_ was 0.2 nm) for CAIX, and more than 10‐thousand‐fold selectivity over CAI and CAII, the two most important cytosolic isozymes responsible for maintaining the blood physiological pH [32]. High‐affinity compound for the target protein is important for optimal tumour imaging, as it delays clearance of the compound—by using GZ22‐4 probe, even after 48 h we could obtain a very clear tumour image. Noteworthy, our study demonstrated that the GZ22‐4 probe, initially designed for human CAIX detection, is able to detect murine CAIX and thus is well suited also for preclinical test systems involving murine cancers. This is in line with the fact that the highest sequence similarity between human and murine CAIX is found in the catalytic domain of the protein [13].

### Description of the *in vivo* study observations and macroscopic findings

**Fig. 4:**
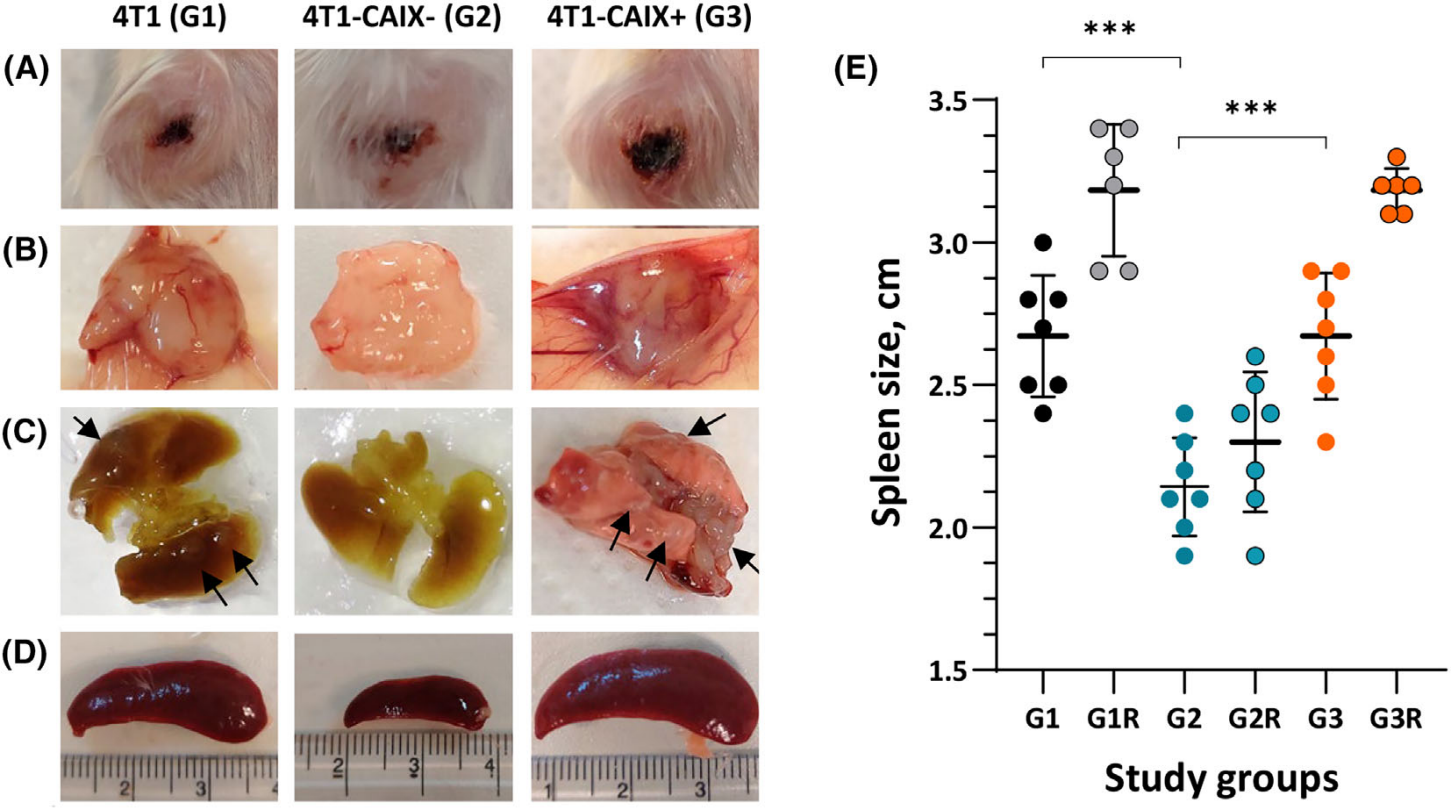
Macroscopic evidence of mCAIX expression‐associated changes in primary tumours, lung metastases, and spleen size. (A) Representative examples of ulcerative lesions of primary tumours at the repeated study endpoint (i.e., day 37 for groups 1 and 2, and day 31 for group 3). (B) Macroscopic appearance of extirpated primary tumours at study endpoint showing gross differences in vascularisation. (C) Representative examples of lung metastatic lesions at the repeated study endpoint; black arrows indicate macrometastases. (D) Comparison of spleen size between study groups at the repeated study endpoint (representative examples). (E) Comparison of spleen size between study groups in the exploratory and repeated study (mean, SD); dots represent individual animals (*n* = 6–7). Data for the exploratory study groups were compared (one‐way ANOVA multiple comparison‐adjusted *P*‐values shown). 4T1‐CAIX^−^, 4T1 cell line with no mCAIX production; 4T1‐CAIX^+^, 4T1 cell line with constitutive mCAIX production; G, group; R, replicate; ***P = 0.0001.

In the exploratory study, animals did not show any signs of suffering till the experiment termination, and no significant weight loss was observed in either of the study groups (data not shown) suggesting that tumour volumes may be allowed to increase in the experiments with a therapeutic setting. Nevertheless, two animals from the 4T1‐CAIX^+^ group had to be euthanized on day 20 due to progressive non‐haemorrhagic ulcers with present necrotic areas developing on the primary tumour surface (Fig. 4A). On average, 4–5 animals out of 7 in each group developed signs of ulceration at the nipple area of the tumours irrespectively of the study group in the last 4–6 days before the endpoint (Table 1, Fig. 4A). However, from the animal observation and previously reported data, it was concluded that most of the necrotic lesions most likely originated from tissue damage caused by self‐induced trauma (itching‐scratching) most likely initiated by the rapid tumour growth *per se*.

However, in the repeated study, due to later endpoints, the necrotic lesions were more prevalent (Table 1) indicating the direct correlation of the necrotic lesion incidence with the developing tumour size. This should be taken into account when designing therapeutic intervention studies in this model system since it will directly affect the dropout rate of study animals and the set outcome measures. Tumour weight closely positively correlated with the tumour volume at the study endpoint (Spearman *r* = 0.8; 95% CI: 0.65–0.89, *P* < 0.0001; data combined from both studies, *n* = 42).

The mice bearing mCAIX‐expressing tumours demonstrated pronounced splenomegaly (Table 1 and Fig. 4D,E), a well‐known feature of the 4T1 mouse model [32], and the spleen length positively correlated with the primary tumour weight in animals with 4T1 tumours (Spearman *r* = 0.71, 95% CI: 0.24–0.91, *P* = 0.0043) and 4T1‐CAIX^+^ tumours (Spearman *r* = 0.79, 95% CI: 0.42–0.94, *P* = 0.0009). Nevertheless, the mice from the 4T1‐CAIX^−^ group had normal spleen size in both studies (see Table 1, Fig. 4D,E) irrespective of the primary tumour size (Spearman *r* = 0.26, 95% CI: −0.33 – 0.70, *P* = 0.19;). The difference between the study groups was statistically significant (one‐way Kruskal–Wallis *P* = 0.0004; Fig. 4E).

Splenomegaly in 4T1 tumour‐bearing mice is correlated with a significant increase in immature splenic granulocytes [40, 41]. Cultured 4T1 cells have been shown to constitutively express myeloid colony‐stimulating factor (CSF), and in syngeneic mice, the level of 4T1 cancer cell‐derived G‐CSF correlates with splenomegaly as demonstrated by Yoshimura *et al*. [41]. To the best of our knowledge, the direct mechanistic link between the increased tumour‐associated CAIX production and G‐CSF‐mediated splenomegaly has not been established. However, Chafe and colleagues have demonstrated that CAIX is a key player of pH‐dependent NF‐κΒ activation that results in elevated G‐CSF levels aiding in the premetastatic niche establishment [42] thus providing a rationale behind our finding in the 4T1‐CAIX^−^ group. Taken together, the data obtained in our study have convincingly shown that loss of mCAIX production in 4T1 tumours results in the preservation of normal spleen size (i.e., prevention of splenomegaly) in the tumour‐bearing syngeneic mice. Further research is warranted to prove the causality and gain mechanistic insight into this finding.

### Histological evaluation of the primary tumours

**Fig. 5:**
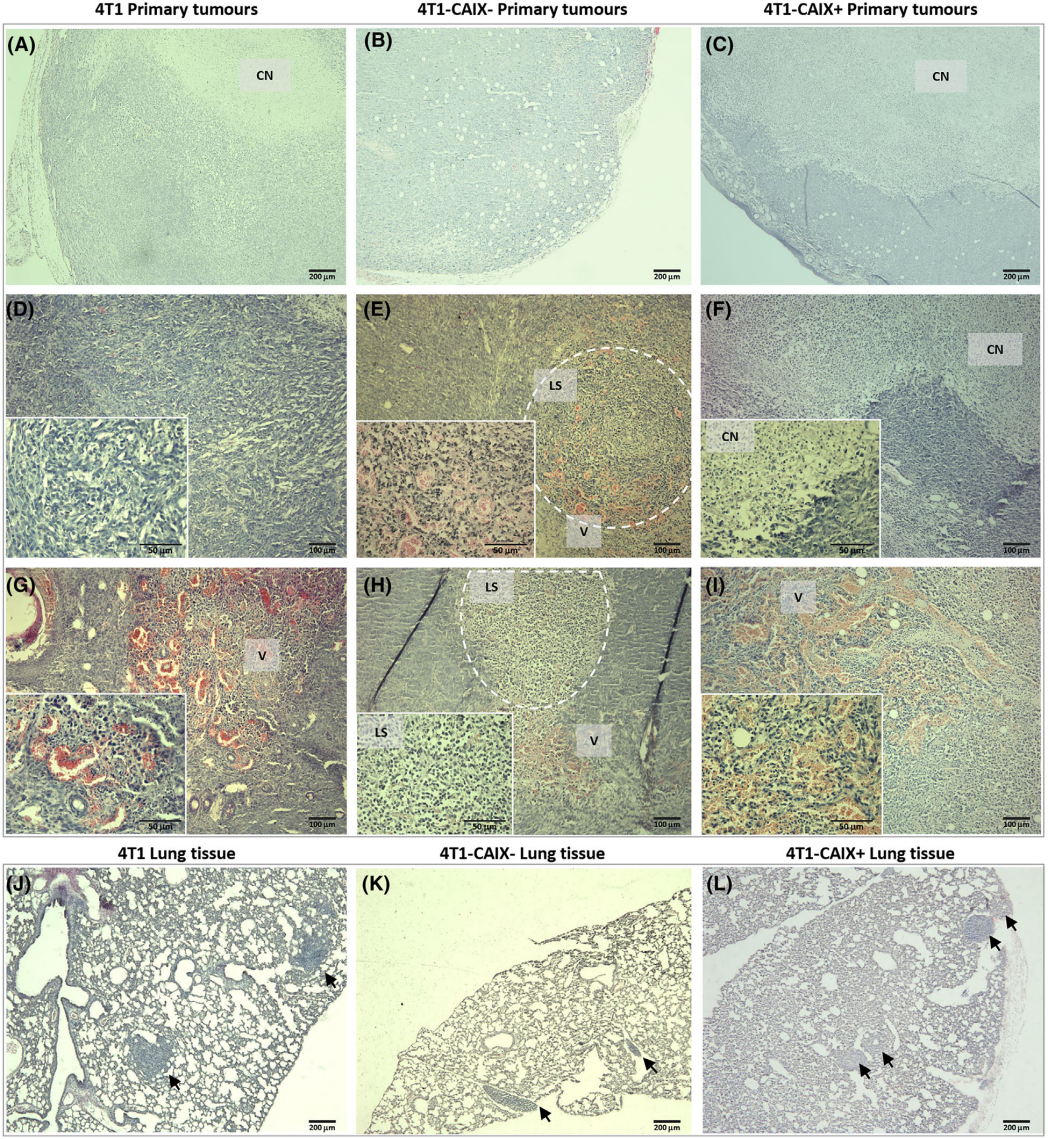
Histological representation of the developed 4T1 breast cancer primary tumours and lung metastases. Upper panel (A–I): representative microphotographs of primary tumour tissue sections from the three study groups, stained with H&E. (A–C) Cross‐sectional view of the primary tumours demonstrating areas of coagulative necrosis (scale bar—200 μm). (D–I) Differential features of the primary tumour tissues (main panel scale bar—100 μm, inner panel scale bar—50 μm). Lower panel (J–L): H&E staining of lung tissue sections harvested from tumour‐bearing mice at study endpoint [i.e., day 26 after tumour cell injection for animals bearing 4T1 (J) and 4T1‐CAIX^+^ tumours (L), and day 39 after 4T1‐CAIX^−^ tumour cell injection (K); scale bar—200 μm, the black arrows indicate micrometastases]. 4T1‐CAIX^−^, 4T1 cell line with no mCAIX production; 4T1‐CAIX^+^, 4T1 cell line with constitutive mCAIX production; CN, coagulative necrosis; LS, lymphoid structures; V, vasculature.

Macroscopic examination of the developed primary mCAIX‐expressing tumours showed that they were systematically displaying a more vascularised phenotype in comparison to the mCAIX‐deficient tumours (see Fig. 4B). This observation was confirmed by histological examination that showed pronounced structures with leaky vascularisation in the periphery of tumours (Fig. 5G,I) while the centrally located areas of CAIX‐expressing primary tumours were poorly vascularized and systematically demonstrated extensive coagulative necrosis (see Fig. 5A,C,F for representative examples). These necrotic areas were surrounded by peripheral proliferative tumour regions (Fig. 5D).

Coagulative necrosis is a typical characteristic of 4T1 tumours [16, 43] and it is most commonly caused by hypoxic–ischemic conditions and high lactate production [44]. This type of necrosis has been linked with worse prognosis in solid malignancies including invasive breast cancer [45, 46]. CAIX production has been associated with tumour necrosis and vascularisation in pancreatic cancer [47] and bladder cancer [48] and thus our data are in agreement with the existing knowledge on the correlation of CAIX production with the necrosis development—such necrotic regions were not observed in the 4T1‐CAIX^−^ tumours that exclusively demonstrated homogeneous proliferative tumour histology (Fig. 5C). Noteworthy, in the proximity of blood vessels, immune cell infiltration was frequently observed. In the case of CAIX‐positive tumours, the leukocyte infiltration systematically showed a diffuse pattern while in the 4T1‐*Car* ^KO^ tumours, the formation of more organised and lymphatic nodule‐like structures was observed in several cases (Fig. 5E,H). It has been shown that extracellular tumour microenvironment acidification resultant from the CAIX upregulation and activity possesses a barrier for lymphocyte homing in tumour tissues and that upon CAIX depletion, increased CD3+ T cell infiltration is observed in a murine melanoma model [49].

### Description of the metastatic lesion findings

Since the tumour growth dynamic was set as the primary outcome measure in the performed *in vivo* studies, systematic assessment and comparison of the metastasis development could not be performed due to differences in study endpoints between the study groups. Yet, an indirect relative comparison of the lung metastases burden can be performed if comparing groups 1 and 3 from the exploratory study and groups 1 and 2 from the repeated study (Table 1). On day 25/26 after tumour cell implantation, no observable macrometastases were detected in groups 1 and 3 animals, although for some mice (2 animals from each group) initiation of lung metastases formation was observed on the lung surface. In the 4T1‐CAIX^−^ tumour‐bearing animals (group 2), for 6 out of 7 animals, observable small to medium‐size metastatic nodules were detected on the lung surface at the study endpoint (day 39 post tumour cell implantation). When examining the lung tissue sections, lung metastases were detected in histological samples from all animals irrespective of the study group (Fig. 5J–L).

In the repeated study, due to later endpoints, the number and size of the metastatic load were significantly increased, demonstrating progressive metastasis development—all animals except for one animal with 4T1‐CAIX^−^ tumour (on day 37) and one with 4T1‐CAIX^+^ tumour (on day 30) had macroscopically detectable lung metastases (Table 1, Fig. 4C). In this study, groups 1 and 2 could be compared—the gross examination of lung metastases load showed that 4T1‐CAIX^−^ tumour‐bearing animals have smaller metastatic nodules in comparison to the animals with 4T1 tumours, indicating slower tumour progression in animals with deficient mCAIX, which is in line with data from other studies exploiting CAIX‐depleted breast cancer cell lines [17, 18, 19]. Indeed, CAIX has been demonstrated to contribute to decreased cell–cell adhesion and increased migration of cancer cells [13]. Apart from lung metastases, occasional extra‐pulmonary metastatic findings were detected in several cases (Table 1).

### Constitutive mCAIX overexpression does not trigger autoantibody responses *in vivo*

Since B cell tolerance is less stringent than T cell tolerance, in many cases the development of antigen‐specific high‐titre IgG responses against autologous overexpressed tumour‐associated antigens (TAAs) has been observed in various types of cancer in humans [50, 51, 52, 53]. CAIX is a well‐known TAA identified in renal cell carcinoma [54]. To assess the presence of anti‐mCAIX IgG autoantibodies possibly elicited by autologous CAIX overexpression, serum samples taken from all study groups at the endpoint were tested against the recombinantly expressed extracellular catalytic domain of mCAIX by using ELISA. Results revealed that the constitutive overexpression of autologous CAIX protein in the 4T1‐CAIX^+^ tumours did not lead to the systematic autoantibody production against the extracellular catalytic domain of murine CAIX—low‐titre anti‐mCAIXc IgG antibody responses (detectable at 1 : 85 serum dilution) were demonstrated only in one out of 14 4T1‐CAIX^+^ tumour‐bearing animals, and two out of 14 mice with parental 4T1 tumours (1:50 and 1:55 serum dilution) while all the animals with 4T1‐CAIX^−^ were anti‐mCAIXc IgG negative. As reported previously, the threshold for defining a positive autoantibody signal against TAAs has been set to at least 1 : 200 serum dilution [50, 51, 52, 53]. Hence, it can be concluded that the model system is fully suitable for one of the potential further applications of the developed model system, namely therapeutic CAIX‐targeted vaccination since there is no induction of specific anti‐mCAIX immune responses observed, which could interfere with the treatment.

### Potential applications of the developed model system

A large body of data has accumulated for the metastatic 4T1 model – it has been used as an experimental animal model for human TNBC for more than 30 years [14], has good face validity, and many advantageous characteristics. First, tumour cells can be easily transplanted into the mammary gland fat pad without the need for surgical intervention—the ease of orthotopic transplantation ensures that the primary tumour grows in the proper tissue microenvironment. Although both orthotopic and subcutaneous 4T1 tumour models in syngeneic hosts have been used for experimental procedures, these have been shown to display differential patterns with regard to vascularisation, tumour growth, and metastasis rates [27] thus emphasising the importance of the proper tumour microenvironment as a necessary requirement for increasing the result translatability. The orthotopic tumour proximity to the body surface easily allows for its surgical removal for experimental purposes to resemble the clinical setting used in humans, as well as serves as an excellent feature for *in vivo* imaging. Noteworthy, new CAIX‐specific imaging agents are being developed for non‐invasive screening of solid cancers, diagnostic, and prognostic purposes [21, 22]. Next, 4T1 is a spontaneous metastatic model with a well‐described cancer progression sequence and the involved players, CAIX being among them [16]. Importantly, human CAIX structure and functions have been systematically compared with its murine orthologue mCAIX by Pastorekova's group [13], which revealed high functional similarity of the two proteins including pH regulation, decreased cell–cell adhesion, and increased migration, providing evidence that the role of murine CAIX in cancer development closely mimics that in human cancer, thus strengthening the translational value of mouse models addressing the CAIX role in cancer. Moreover, the 4T1 model is syngeneic and fully immunocompetent, which is a prerequisite for its use for testing immunotherapeutic strategies targeting the activation of tumour‐specific adaptive immunity effectors [14]. Noteworthy, the frequently used human cancer patient‐derived tumour xenograft models do not provide the immune system components necessary for immunotherapy efficacy evaluation, while the murine models with a humanised immune system have inherently higher translatability potential but are rather costly alternatives.

CAIX emerged as a potential cancer target more than three decades ago [55], and the field of CAIX research has advanced significantly, together with the preclinical model portfolio for CAIX research. Despite the progress in the field, novel CAIX inhibitors [56] and imaging agents [21] are still being developed, and the availability of proper preclinical model systems for their evaluation is of great importance. Moreover, CAIX has emerged as an immunotherapy target due to the cell surface expression of its catalytic and proteoglycan‐like domains, both of which have been implicated in cancerogenesis [20]. The well‐characterised therapeutic monoclonal antibody G250 recognising native cell surface CAIX in renal cell carcinoma is one of the most visible examples of CAIX immunotargeting [57]. The developed orthotopic 4T1 mouse model system is immunocompetent, syngeneic, and autologous with regards to the produced CAIX, and thus ensures full competence in terms of the natural tumour microenvironment and immunological responses necessary for the assessment of immune responses elicited by both, the immunotherapy and the tumour development itself. The introduction of positive and negative controls for mCAIX expression enables a more reliable interpretation of the CAIX‐targeted therapy efficacy and thus may significantly contribute to the proof‐of‐principle. Moreover, this model system can be applied for combining immunotherapy with other CAIX‐targeted therapies or conventional treatment modalities like chemotherapy, radiotherapy, or surgery. However, we acknowledge a limitation in the characterisation of the model system as it lacks controls for potential effects associated with the genetic editing of 4T1 cells. As a result, certain CAIX‐independent effects—such as those related to Cas9 expression or puromycin selection—may not have been fully accounted for.

In this study, we provide a detailed description of the applied methodology for the model system development as well as describe relevant model system characteristics, which enable a better definition of time points for planned therapeutic intervention setting and more precise calculation of sample size for further studies, considering the expected animal dropout rates. We have demonstrated that the developed model system is robust and can be well repeated *in vivo* and most likely could be reproduced in other research laboratories and thus represents a valuable addition to the existing CAIX model system portfolio.

## Conflict of interest

DM, AZ, and EC declare that they have patent applications and patents on Carbonic Anhydrase inhibitors, including GZ22‐4. The rest of the authors declare that they have no known competing financial interests or personal relationships that could have appeared to influence the work reported in this paper.

## Peer review

The peer review history for this article is available at https://www.webofscience.com/api/gateway/wos/peer‐review/10.1002/2211‐5463.70052.

## Author contributions

ZK, KT, and DM conceptualised and designed the study. ZK, IL, SK, RP, GŽ, AP, AZ, MTL, VS, JJ, MK, and EČ performed the experiments. ZK, SK, VS, MTL, JJ, GŽ, and AP acquired, analysed, and interpreted the data. ZK and GŽ prepared the figs; ZK wrote the original manuscript draft. KT, GŽ, AP, and DM reviewed and edited the manuscript. ZK, KT, and DM supervised the project and secured funding. All authors read and approved the final manuscript.

## Data Availability

Data associated with this study has not been deposited into a publicly available repository and will be made available on request.
